# Determinants of health outcome in individuals with asthma

**DOI:** 10.1186/2045-7022-3-S1-P19

**Published:** 2013-05-03

**Authors:** Malin Axelsson, Linda Ekerljung, Bo Lundbäck, Jan Lötvall

**Affiliations:** 1 Gothenburg University, Sahlgrenska Academy, Institute of Medicine, Krefting Research Centre, Sweden

## Background

For many individuals, asthma has great impact on their everyday life, not least in terms of medication treatment and disease management. Therefore, it is important to evaluate recommended treatments on an individual level. In that respect, estimations of health-related quality of life (HRQL) function as an essential health outcome, as they capture personal perspectives and experiences of everyday life with a chronic disease such as asthma. The aim was to identify determinants of health-related quality of life in adult individuals with asthma.

## Method

Participants with asthma (n=487) derived from a population-based study completed questionnaires on asthma control, emotional status, adherence and HRQL. Additionally, data on the Five-factor model personality traits: neuroticism, extraversion, openness, agreeableness, conscientiousness were gathered. Data were statistically analyzed using t-tests, bivariate correlations and multiple regressions.

## Results

Participants, who perceived their asthma as being poorly controlled (n=126, 26.3%), reported both poorer emotional status and HRQL. Participants with poor physical HRQL seemed to have both poorer emotional status and asthma control. Moreover, participants with low levels of Extraversion were more likely to perceive their physical HRQL as worse. Participants with poor mental HRQL reported poorer emotional status and adherence to asthma medication as well as poorer asthma control. Additionally, participants with high levels of neuroticism and low levels of extraversion, agreeableness and/or conscientiousness perceived their mental HRQL as worse. Asthma control was identified as predictor for physical HRQL. Emotional status, adherence to asthma medication, asthma control, neuroticism and agreeableness were identified as predictors for mental HRQL.

## Conclusion

The current findings argue that in addition to medical examinations, we also need to consider asthmatics emotional status, adherence behavior, asthma control but also personality characteristics in evaluations of their health outcome.

